# The damage-associated molecular pattern cellotriose alters the phosphorylation pattern of proteins involved in cellulose synthesis and *trans*-Golgi trafficking in *Arabidopsis thaliana*

**DOI:** 10.1080/15592324.2023.2184352

**Published:** 2023-03-13

**Authors:** Akanksha Gandhi, Yu-Heng Tseng, Ralf Oelmüller

**Affiliations:** Matthias Schleiden Institute of Genetics, Bioinformatics and Molecular Botany, Department of Plant Physiology, Friedrich-Schiller-University, Jena, Germany

**Keywords:** Cell wall, protein phosphorylation, Arabidopsis, membrane protein trafficking, malectin domain

## Abstract

We have recently demonstrated that the cellulose breakdown product cellotriose is a damage-associated molecular pattern (DAMP) which induces responses related to the integrity of the cell wall. Activation of downstream responses requires the Arabidopsis malectin domain-containing CELLOOLIGOMER RECEPTOR KINASE1 (CORK1)^1^. The cellotriose/CORK1 pathway induces immune responses, including NADPH oxidase-mediated reactive oxygen species production, mitogen-activated protein kinase 3/6 phosphorylation-dependent defense gene activation, and the biosynthesis of defense hormones. However, apoplastic accumulation of cell wall breakdown products should also activate cell wall repair mechanisms. We demonstrate that the phosphorylation pattern of numerous proteins involved in the accumulation of an active cellulose synthase complex in the plasma membrane and those for protein trafficking to and within the *trans*-Golgi network (TGN) are altered within minutes after cellotriose application to Arabidopsis roots. The phosphorylation pattern of enzymes involved in hemicellulose or pectin biosynthesis and the transcript levels for polysaccharide-synthesizing enzymes responded barely to cellotriose treatments. Our data show that the phosphorylation pattern of proteins involved in cellulose biosynthesis and *trans*-Golgi trafficking is an early target of the cellotriose/CORK1 pathway.

## Introduction

The shape of a plant cell is determined by its wall, and its integrity is essential for all wall-containing cells. Growth, differentiation, maintenance of the turgor, tropisms, and many other developmental processes require alterations in the cell wall architecture that are orchestrated by signals originating from the plant itself.^2–5^ Furthermore, the cell wall is the barrier to the environment, and has to cope with abiotic and biotic stresses. Drought, salinity, or toxic compounds force the cells to induce adaptive or compensatory changes, which maintain their architecture and protect the cell; beneficial and pathogenic microbes, insects, and nematodes or physical damage destroy cell wall material, which must be repaired.^2,3,6,7^ Therefore, cells have developed perception and signaling mechanisms through which they respond to damage of their walls.

The major polysaccharides in the plant cell wall are cellulose, hemicellulose, and pectin. Hemicellulose and pectin are made inside the cell at the Golgi apparatus by the coordinated action of many proteins^8^ while cellulose is synthesized at the plasma membrane by the cellulose synthase (CESA) complex (CSC).^9^ The elongating cellulose molecules assemble in the apoplast to form microfibrils with a paracrystalline structure, which associates with hemicellulose, pectin and apoplastic proteins. Breakdown products of the cell wall polysaccharides act as damage-associated molecular patterns (DAMPs;^10^) and are recognized by cell surface receptors. Breakdown products of pectin are oligogalacturonides (OGs) which activate the Wall-Associated Kinase 1 (WAK1).^11,12^ In rice, mixed-linked β-1,3/1,4-glucans from hemicellulose breakdown, namely 3^1^-β-D-cellobiosyl-glucose and 3^1^-β-D-cellotriosyl-glucose, bind to CERK1, and induce the dimerization of CERK1 and the rice chitin receptor CEBiP.^13^ Cellooligomers from cellulose breakdown require the malectin-domain (MD) receptor kinase (RK) CORK1 for intracellular signaling.^1,14^ Therefore, breakdown products of the three cell wall polysaccharides are recognized by pattern recognition receptors (PRR)s.

Here, we investigate early responses which are induced by cellotriose in the roots of wild-type and *cork1* mutants. Cellulose degradation products (cellooligomers) are generated by plant and microbial enzymes and Aziz et al.^15^ has already shown that they induce a variety of defense responses in grapevine (*Vitis vinifera*) cells. Locci et al.^16^ showed that cellotriose and, to a lesser extent, cellotetraose to cellohexose, induce ROS production, phosphorylation of MAPKs and other proteins, as well as the activation of defense gene expression. Souza et al.^17^ demonstrated that cellobiose triggers a signaling cascade that shares similarities to responses to well-known elicitors such as chito-oligomers and OGs. In contrast to other known P/DAMPs, cellobiose stimulates neither ROS production nor callose deposition. Transcriptome profiles are very similar after cellobiose and OG treatments.^17,18^ Johnson et al.^19^ showed that cellotriose, induces rapid cytoplasmic Ca^2+^ elevation in Arabidopsis and tobacco root cells. It acted synergistically with chitin. Induction of the Ca^2+^ response by cellotriose and activation of the downstream responses requires the poly(A) ribonuclease (AtPARN; At1g55870) which degrades the poly(A) tails of specific mRNAs in roots. Comparison of cellooligomers of different lengths demonstrated that cellotriose is the most active cellooligomer for the induction of downstream responses.^19^ Thus, evidence for cellooligomer-induced signaling in plants have been reported for several systems. More recently, Tseng et al.^1^ and Martin-Dacal et al.^2014^ identified the MD containing LRR receptor kinase CORK1 as cellotriose receptor in Arabidopsis.

Cellotriose/cellobiose application to Arabidopsis roots and shoots has profound effects on the expression profile^17^,^19^ and phosphoproteome pattern in Arabidopsis roots.^1^ Besides triggering calcium influx, ROS production, electrochemical potentials across the plasma membrane, mitogen-activated protein kinase (MAPK) activation, and defense-related gene expression, previous studies suggest that the cellooligomer application also leads to higher pathogen resistance, similar to observations with pectin and hemicellulose breakdown products.^17,19,21–25^ The defense responses induced by cellooligomers are relatively mild when compared to those induced by the pathogen-associated molecular patterns (PAMPs) chitin or flg22.^12,17,19,23^ However, in combination with chitin, flg22 or the chitin breakdown product oligogalacturonic acid, synergistic effects on calcium influx, ROS production, and MAPK activation indicate crosstalk between cellooligomer, in particular cellotriose, and PAMP responses.^17,19^

MD-RKs and MD-like (MDL)-RKs are encoded by a small gene family in Arabidopsis,^25^ and several members are involved in sensing the integrity of the cell wall. While little is known about MD-RKs, the members of the MDL-RKs are better characterized. The MDL-RK FERONIA, e.g., is involved in monitoring cell wall integrity (CWI) and is required for pollen tube development and plant growth.^26–28^ Its extracellular region interacts with pectin.^29,30^ MDL-RKs also interact with other cell-surface PRRs and intracellular nucleotide-binding leucine-rich repeat receptors (NLRs). Rapid alkalinization factor (RALF) peptide ligands, LORELEI-like glycosylphosphatidylinositol-anchored proteins and cell-wall-associated leucine-rich repeat extensions coordinate with MDL-RKs to orchestrate PRR- and NLR-mediated immunity.^31^ The requirement of the MD-RK CORK1 for many cellooligomer-induced cellular responses suggests that this PRR is also involved in CWI signaling.

An obvious response to impaired cellulose microfibrils should be the activation of CESA to stimulate cellulose repair. Cellulose, a polymer of long unbranched β-1,4-linked glucan chains, is synthesized by CSCs, which are assembled in the Golgi and secreted to the plasma membrane through the *trans*-Golgi network (TGN) compartment. Transport occurs in small CESA compartments (called SmaCCs) or microtubule-associated CESA compartments (called MASCs), which appear to be specific for CESAs and differ from vesicles involved in sorting and trafficking of other cargos. Six CESA heterotrimers, i.e. 18 CESA proteins, constitute the CSC, a high-order oligomer which can be visualized in scanning electron microscopy as sixfold symmetrical rosettes.^32–35^ CESA proteins constitute the catalytic core of this complex, and the newly synthesized glucan chains are directly released into the apoplast.^36^ Cellulose synthesis requires the fully assembled CSC at the plasma membrane, and the complex is stabilized in the plasma membrane by conserved regions and helical exchanges within the transmembrane segments of the individual CESAs.^37^ CSC forms three channels that are occupied by nascent cellulose polymers. Secretion of the chain into the apoplast steers the polymers to a common exit point which may facilitate protofibril formation. The N-terminal domain of the CESAs assembles into a stalk at the cytoplasmic site of the membrane, which allows interaction with microtubules and associated proteins.^37,38^ Amino acids in the N-terminal segment are major targets for phosphorylation,^34,37–42^ ubiquitination,^43^ or acylation,^44^ which ensure proper exocytosis of the complex to the plasma membrane and its recycling via clathrin-dependent endocytosis.

We describe here that the phosphorylation pattern of CESAs, proteins involved in the CESA exocytosis and those facilitating trafficking from the Golgi apparatus to the plasma membrane are early targets of cellotriose signaling. Alterations in the phosphorylation pattern are also observed for proteins involved in CESA internalization from the plasma membrane via clathrin-dependent endocytosis. Since the phosphorylation pattern of enzymes involved in the biosynthesis of hemicellulose and pectin is barely changed, cellotriose appears to trigger preferentially cellulose biosynthesis.

## Materials and methods

### Phosphoproteomic analysis

The Arabidopsis *cork1-2* insertion mutant line (N674063; SALK_021490C) was obtained from Nottingham Arabidopsis Stock Center (NASC). Homozygous seedlings were crossed to the Columbia wild-type line pMAQ2. The corresponding segregated wild-type and homozygous seedlings from the F3 generation were used for experiments, as earlier described in Tseng et al.^1^ For the phosphoproteome analysis, cellotriose (or water, as control) was applied to 300 roots from the segregated wild-type and homozygous F3 seedlings at 0 min, or after treatment with either water or 10 µM cellotriose for 5 or 15 min. Samples were immediately frozen in liquid nitrogen until phosphoproteome analysis, as described in detail in.^1^ The mass spectrometry proteomics data have been deposited to the ProteomeXchange Consortium via the PRIDE partner repository (http://www.ebi.ac.uk/pride) with dataset identifier PXD033224. Data are based on three independent experiments and the statistical analysis is shown in the deposited datasets.

### Transcriptome analysis

Expression profiles were obtained with roots 1 h ^1^, 4 h, or 8 h ^19^ after cellotriose application, water was used as control. RNA hybridization was performed according to Agilent’s One-Color Microarray-Based Gene Expression Analysis (cf.^19^). The accession numbers are given in the publication [for the 1 h time point in^1^ and the other time points in^1^]. Statistical tests were performed using R Studio v1.1.463 with R v4.1.2.

## Results and discussion

### Cellotriose changes the phosphorylation pattern of CESAs

Genes for 10 CESA isoforms are present in the Arabidopsis genome.^45^ Three distinct CESA proteins are necessary to form a functional complex: CESA1, CESA3, and CESA6-like proteins (either CESA2, −5, −6 or −9) are required for primary cell wall synthesis, whereas CESA4, CESA7, and CESA8 are required for secondary cell wall synthesis in Arabidopsis.^46–48^ The amino acid sequences of the CESAs diverge within their N-terminal cytoplasmic domains which are the major targets for regulation (cf. Introduction). For instance, the well-investigated CESA5 is phosphorylated at four positions in its N-terminal region (Ser122, Ser126, Ser229, and Ser230).^49^ In our study, cellotriose application altered the phospohorylation pattern of CESA1 and −3 at various serines in their N-terminal cytoplasmic segments (Table 1). After 5 min, CESA1/Ser152 and CESA3/Ser176 were phosphorylated, whereas CESA3/Ser151/6 was dephosphorylated, while all analyzed serines in the two CESAs were dephosphorylated 15 min after cellotriose application. Although many phosphorylation sites in the CESAs are conserved across the plant species,^49^ to our knowledge, these phosphorylation sites have not yet been described (cf.^49^). We also observed phosphorylation of CESA4/Ser93 after 15 min, but the results were not significant (http://www.ebi.ac.uk/pride). Since CESA4 was the only detected CSC isoform for cellulose synthesis of the secondary cell wall, cellotriose signaling appears to target primarily CESAs for cellulose of the primary cell wall. The (de-)phosphorylation events at the three CESAs differed for at least one time point between wildtype and *cork1* roots: e.g., while all serines are dephosphorylated 15 min after the stimulus in the wild-type, this was not observed in the *cork1* mutant. This suggests that these phosphorylation events are controlled by cellotriose signaling via CORK1.

**Table 1.** Proteins for or associated with cellulose biosynthesis and/or cellular protein sorting which are differentially phosphorylated in Arabidopsis wild-type and *cork1-2* mutant roots 5 or 15 min after cellotriose application. The table also shows the changes of the phosphorylation pattern of the respective amino acids 5 or 15 min after the cellotriose stimulus in wild-type roots relative to *cork1-2* roots. The amino acid positions are shown. For detailed description, cf. text. The mass spectrometry proteomics data have been deposited to the ProteomeXchange Consortium via the PRIDE partner repository (http://www.ebi.ac.uk/pride) with dataset identifier PXD033224 (cf. also^1^ for the identification method). 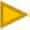, significant; 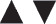, non-significant; 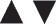 , not (de)-phosphorylated. CORK1-2, insertion line SALK_021490C, N674063), detailed information is provided in.^1^ For significance analysis, cf. deposited datasets.

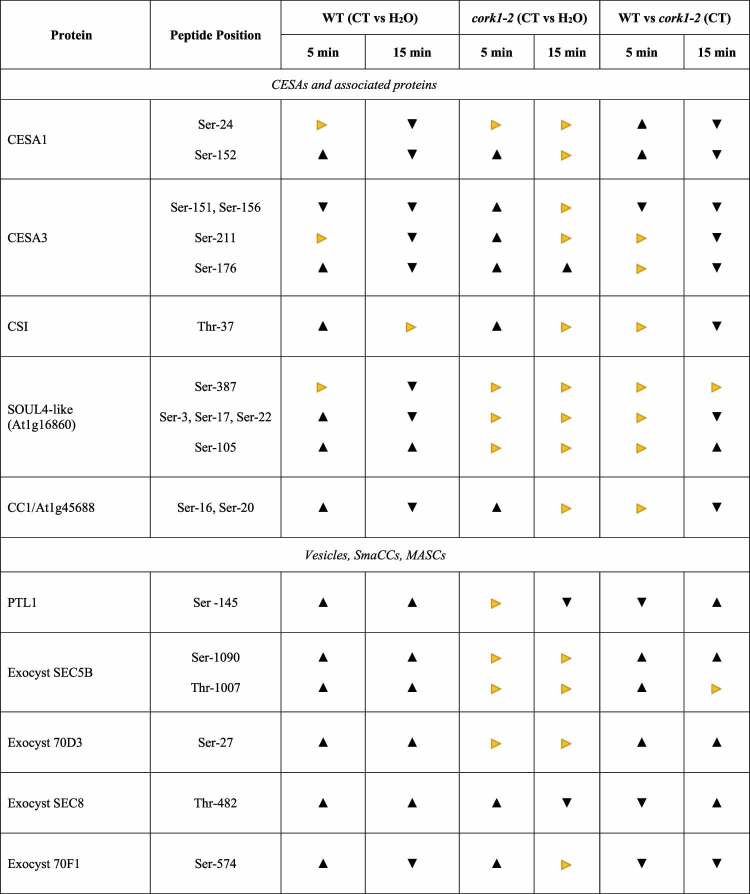
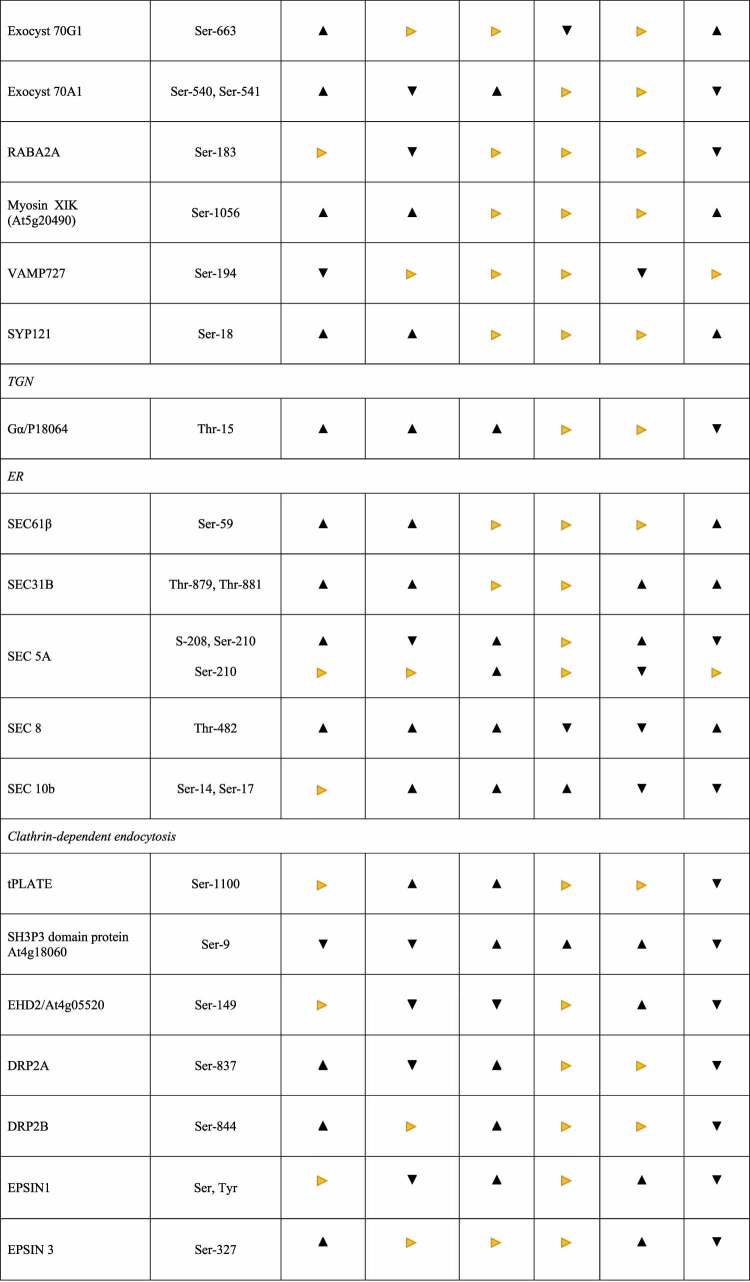
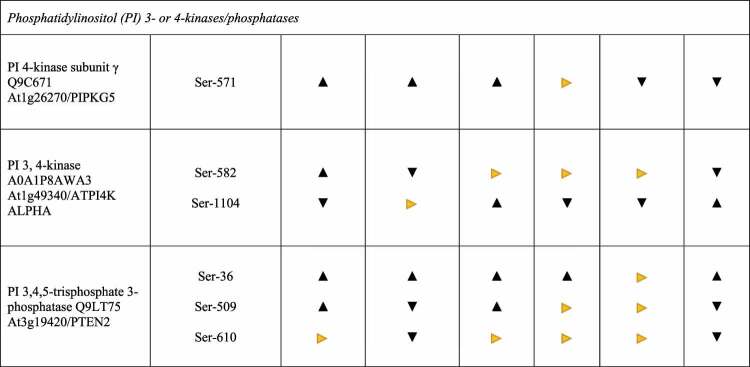

CESA phosphorylations have different effects and occur in response to different stimuli. Mutations of CESA1 phosphorylation sites modulate cell expansion and mobility of CSC.^40^ Genetic studies of CESA1 showed that phosphorylation of Thr166, Ser686, or Ser688 is necessary for tethering between CESA1 and cortical microtubules, whereas phosphorylation of Ser162, Thr165, or Ser167 weakens or impairs this interaction, implying differential regulation of interactions of CESA1 with accessory proteins and thus plasma membrane integration.^36^ Furthermore, the phosphorylated Ser162 can down-regulate cellulose biosynthesis as it primes the surrounding phosphorylation sites for inhibitory phosphorylation by Brassinosteroid Insensitive 2 (BIN2) protein kinase involved in brassinosteroid signaling.^42^ Therefore, BIN2, together with other (a) kinase(s), negatively regulates cellulose synthesis by phosphorylating CESA1, consistent with the observation that brassinosteroids inhibit root growth and cell elongation in Arabidopsis.^42^ Bischoff et al.^39^ showed that phosphorylation of primary cell wall CESAs is induced by phytochrome. CESA7 phosphorylation causes its degradation via a 26S proteasome-dependent pathway.^50^ These examples demonstrate that CESA phosphorylations can either promote or inhibit its plasma membrane integration and thus potentially cellulose biosynthesis activity. A comparative analysis of the phosphorylation events at the different amino acids in response to different stimuli may help to define the fate of the phosphorylated CESAs, and to identify the kinases/phosphatases involved in the regulatory circuits.

### Cellotriose changes the phosphorylation pattern of proteins interacting with CSC at the plasma membrane

CESA-Interactive Protein 1 (CSI1) interacts with CESAs and the cortical microtubules^51^ and appears to have multiple functions: (a) It participates in the delivery of CSCs from the cortical microtubules to the plasma membrane. During exocytosis, CESA and CSI1 appear first at the plasma membrane, followed by tethering of CSC-containing vesicles to the plasma membrane, which is accompanied by the appearance of Sec5B, an exocyst subunit, and PATROL1.^52,53^ Zhu et al.^52^ proposed that CSI1 plays a role in marking the docking site of CSCs-containing vesicles to the plasma membrane. (b) CSI controls CSC motility at the plasma membrane. CSI1 is still associated with the CSC after its insertion into the plasma membrane, and CSC motility is reduced in the *csi1* mutant.^54^ (c) Under stress, CSC endocytosis generates SmaCCs or MASCs. They are involved in recycling of CSC from and fast recovery of CESAs at the plasma membrane, and these processes require CSI.^55^ Thus, CSI is a crucial player for CESA activity. It is rapidly phosphorylated at Thr37 within 5 min after cellotriose application to Arabidopsis roots (Table 1).

PATROL1 and the exocyst complex determine the rate of delivery of CSCs to the plasma membrane^52^ and PATROL1 is also phosphorylated in response to cellotriose application 15 min after the stimulus (Table 1). The exocyst complex consist of eight subunits (SEC3, SEC5, SEC6, SEC8, SEC10, SEC15, EXO84, and EXO70),^56^ and the subunits SEC5B (At1g21170), SEC8 (At3g10380) and EXO70D3 (At3g14090) are among the phosphorylated proteins 5 and 15 min after the stimulus. At least for the 15 min time point, all phosphorylations are significantly different from the water control and the *cork1* results, indicating again that cellotriose requires CORK1 for signaling (Table 1). EXO70F1 and EXO70A1 are significantly de-phosphorylated after 15 min. Besides delivering CEASs to the plasma membrane,^52^ the exocyst complex is involved in vesicular trafficking, protein (CESA) recycling and consequently numerous growth effects.^57^

Furthermore, Zhang et al.^58^ showed that myosins, in particular myosin XIK, via its globular tail domain (GTD), participates in vesicle tethering during exocytosis through interaction with the exocyst complex. The myosin XIK GTD binds to several exocyst subunits, and inhibition of myosin XIK activity reduced the rate of appearance and lifetime of exocyst complexes at the plasma membrane. Myosin XIK associates with secretory vesicles earlier than exocyst and is required for the efficient localization and normal dynamic behavior of exocyst complex at the plasma membrane tethering site. Already in 2019, Zhang et al.^59^ showed the importance of these myosins in cellulose production at the cytoskeleton-plasma membrane-cell wall nexus. Myosin XIK is rapidly phosphorylated 5 and 15 min after the cellotriose stimulus and this requires CORK1 (Table 1).

Finally, the small GTPase RABA2A recruits SNARE proteins to regulate the secretory pathway in parallel with the exocyst complex.^60^ The RABA2A-SNARE- and exocyst-mediated secretory pathways are largely independent, and probably select different cargos. CESA transport was not investigated in this study, but it is believed that CESAs utilize primarily the exocyst pathway. RABA2A is dephosphorylated 15 min after the cellotriose stimulus. Again, the cellotriose effect requires CORK1 (Table 1). The Vesicle-Associated Membrane Protein 727 (VAMP727, At3g54300) and the Syntaxin121 (PENETRATION1/ PEN1) (At3g11820) are recruited by or interact with RABA2A^60^. VAMP727 is also dephosphorylated, but Syntaxin121 is phosphorylated after cellotriose application, in a CORK1-dependent manner. Since syntaxin 121 is also involved in other secretory processes (cf.^61^), its specific role in the two secretory pathways is not clear. The opposite phosphorylation patterns of components of the exocyst pathway and RABA2A suggest that cellotriose triggers the first and inhibits the second one although this requires further investigation.

Several additional proteins have been identified to be components of or associated with CSCs at the plasma membrane: The endoglucanase KORRIGAN1 is required for cellulose synthesis by acting as a cellulase at the plasma membrane–cell wall interface. Mutant analysis of this protein showed altered cellulose content in both the primary and secondary cell wall.^62^ The COMPANION OF CELLULOSE SYNTHASE1 (CC1) and CC2 play a role in localizing CESA to the membrane and controls microtuble dynamics.^63,64^ The glycosyl phosphatidylinositol (GPI)-anchored COBRA facilitates cellulose crystallization from the emerging β1–4-glucan chains by acting as a “polysaccharide chaperone”.^65^ Furthermore, the two plasma membrane-localized proteins SHOU4 and SHOU4-like directly interact with CESAs and negatively affect CSC exocytosis.^66^ Finally, TRANVIA (TVA) facilitates trafficking of CSCs to the plasma membrane, and *tva* mutants have defects in CSCs secretion and activity at the plasma membrane.^38^ From these seven proteins, only the phosphorylation pattern of SHOU4-like and CC1 were altered after the cellotriose treatment. This is interesting, since also Korrigan and CC2 has been shown to be phosphorylated during different developmental processes.^49^ Two of the three identified phosphorylation sites in SOUL4-like are significantly dephosphorylated (15 min) and one significantly phosphorylated (5 min) by the cellotriose/CORK1 pathway (Table 1). How this affects the inhibitory effect of SOUL4-like on CESA exocytosis, remains to be determined.

### Protein translocation into the endoplasmic reticulum (ER) and to the trans-Golgi network (TGN)

The first step in the cotranslational translocation of proteins traveling through the ER-secretion pathway is targeting and attachment of the nascent chain to the ER membrane via interaction between the signal sequence and the signal recognition particle and its receptor.^67^ The main component of this complex is the Sec61 protein consisting of α, β, and γ subunits. Oligomers of the Sec61 complex form a transmembrane channel where proteins are translocated across and integrated into the ER membrane. Interestingly, the Sec61 subunits are also observed in the post-ER compartment suggesting that they also play a role in the TGN. Phosphorylation of the β subunit of the SEC61 complex (At5g60460) in wild-type, but not *cork1* roots suggests that translocation of preproteins into the ER is rapidly activated by the cellotriose/CORK1 pathway (Table 1). Furthermore, SEC31B is also rapidly phosphorylated 5 and 15 min in response to the cellotriose/CORK1 pathway (Table 1). SEC31B is involved in the export of cargo from the ER to mobile Golgi stacks.^68,69^ Rapidly phosphorylation of SEC61β and SEC31B suggests that the cellotriose/CORK1 pathway controls early steps in the ER translocation machinery. However, whether this affects also CESAs is not known.

### CESA-specific proteins in the TGN

CSC assembles in the Golgi apparatus, and STELLO1 and STELLO2^70^ and the small GTPase RabH1B^70^ are specifically involved in CESA trafficking via the TGN. STELLO1 and STELLO2 are glycosyltransferases that interact with CESAs in the Golgi lumen, and *stello1/2* mutants are impaired in the spatial distribution within the Golgi, secretion and activity of the CSCs.^70^ Rab-H1b, a small GTPase, participates in the trafficking of CESA6 from the TGN to the plasma membrane.^71^ None of these three proteins are phosphorylated after cellotriose application within 15 min. Either they are not targets of the signaling pathways or not accessible by kinases/phosphatases in the Golgi vesicles. It has been proposed that phosphorylation mainly modulates the activity of CSCs in the plasma membrane rather than having an effect on CESA’s subcellular location or trafficking.^39,40,42,49^ Our phoshoproteome data do not provide evidence in favor or against this postulation, but demonstrate that mainly proteins associated with the integration of CSC into and mobilization at the plasma membrane are phosphorylated after cellotriose application.

Recently, McFarlane et al.^35^ identified a family of seven transmembrane domain-containing proteins (7TMs) that are important for cellulose production during CWI stress. 7TMs are associated with guanine nucleotide-binding (G) protein signaling. Unexpectedly, two members of the 7TMs, 7TM1 and 7TM5, localized to the Golgi/TGN where they interacted with G protein components. The authors showed that 7TMs and Gβγ regulated specifically CESA trafficking but did not affect general protein secretion. They hypothesized that the G protein complex could potentially sense the cell wall status via association with receptor-like kinases at the plasma membrane and regulate CSC secretion via the 7TMs at the endomembrane system. Although we did not identify Gβ in our phosphoproteome analyses, 7TM5 (At2g01070), Gα (At2g26300) and Gγ (At3g22942) are phosphorylated after cellotriose application, although not significantly in comparison to the water control or *cork1* mutant (Table 1 and http://www.ebi.ac.uk/pride).

### Cellotriose signals target the TGN as central trafficking hub

The TGN is a central trafficking hub where secretory, vacuolar, recycling, and endocytic pathways merge.^72^ EPSINs are important players in the TGN vesicle formation. EPSIN1 plays an important role in vacuolar trafficking of soluble cargo proteins via interactions with clathrin and clathrin-associated proteins (cf. below;^73^). More recently, Collins et al.^74^ showed that EPSIN1 also modulates the plasma membrane abundance of the flagellin receptor Flagellin Sensing 2 (FLS2) for effective immune responses. Since the *eps1* mutant is impaired in flg22 signaling and showed reduced plasma membrane accumulation of FLS2 and its coreceptor BRASSINOSTEROID INSENSITIVE1-ASSOCIATED RECEPTOR KINASE1 (BAK1), EPSIN1 also appears to be involved in protein delivery to the plasma membrane. Lee et al.^75^ proposed that other EPSIN members in Arabidopsis might have different functions in protein sorting. EPSIN1 is de-phosphorylated 15 min after cellotriose application, and EPSIN2 and EPSIN3 are phosphorylated 5 and 15 min after the stimulus, and the results for the three proteins are significantly different from the *cork1* control (Table 1 and http://www.ebi.ac.uk/pride). Although the role of these EPSIN phosphorylations for protein sorting and trafficking of CESA-containing vesicles is not or not well investigated, they are targets of cellotriose/CORK1 signaling. It appears that the signaling path interferes with the dynamics of protein sorting in the TGN.

### Endocytosis, cellulose synthase recycling

Like many PRRs, the CSC is internalized from the plasma membrane via clathrin-dependent endocytosis, although a clathrin-independent pathway has been hypothesized as well.^53,76–84^ The function of many proteins in clathrin-mediated endocytosis has been extensively characterized in mammals.^85^ Although many proteins are conserved in plants,^78,82^ only a few components have been associated with CSC endocytosis, recycling, or degradation.

At least 28 proteins have been described to be involved in or associated with clathrin-mediated endocytosis in Arabidopsis,^82^ eight of them are reversibly phosphorylated in response to cellotriose treatment, although to different extents. This includes the two heavy-chain proteins (At3g11130, CHC1) and At3g085330 (CHC2), the SH3 domain protein At4g18060, EHD2 (At4g05520), and Dynamin-Related Protein (DRP)2A (At1g10290), which are all dephosphorylated after 15 min. DRP2B (At1g59610) is phosphorylated at 5 min and the clathrin recruiting tPLATE (At3g01780) as well as the SH3 domain protein At1g31440 15 min after the stimulus (Table 1 and http://www.ebi.ac.uk/pride). From the six types of DRPs encoded in the Arabidopsis genome, two of them (DRP1 and DRP2) participate in post-Golgi trafficking (cf.^86^). In particular, DRP2A and DRP2B function coordinately in multiple pathways of post-Golgi trafficking in a phosphatidylinositol 3- or 4-kinase-dependent manner^86^ and DRP2B plays a role in flg22-signaling and pattern-triggered immunity in plants.^87^ The tPLATE complex is recruited to the plasma membrane during the early stages of endocytosis.^88,89^ The role of the identified phosphorylation changes in response to cellotriose application is not known but comparison of the responses in wild-type and *cork1* roots demonstrates that almost all of them are cellotriose/CORK1-dependent. Furthermore, this appears to be relevant for endocytosis and recycling of the CESAs, since they interact with the Adaptor Protein 2 (AP2)-like and tPLATE complexes.^76,77,88,90^ None of the four main components constituting the AP2 complex^91^,^92^ are phosphorylated in response to cellotriose.

**Figure 1.**
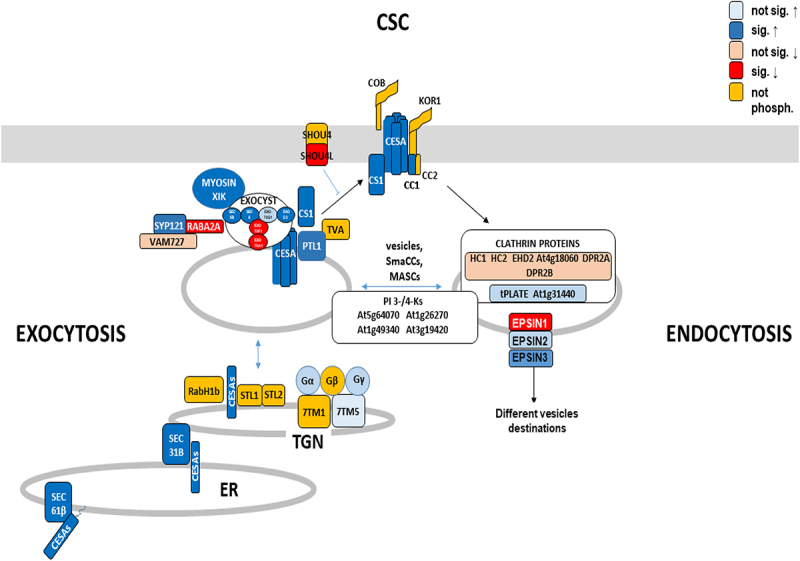
A model showing proteins preferentially involved in CESA exocytosis and endocytosis. Dark (light) blue shows proteins with amino acids which are (not) significantly up-regulated either 5 min or 15 min or at both time points after cellotriose application, the red color shows down-regulated genes. Proteins which do not change their phosphorylation pattern after the cellotriose stimulus are in Orange. For abbreviations and protein names, cf. text.

Huang et al.^86^ demonstrated that post-Golgi trafficking is phosphatidylinositol (PI) 3- or 4-kinase-dependent. Several PI 3- and PI 4-kinases (At5g64070 and At1g26270), the PI 3,4- kinase At1g49340 and the PI 3,4,5-trisphosphate 3-phosphatase (At3g19420) are (de-) phosphorylated 5 or 15 min after the cellotriose stimulus (Table 1 and http://www.ebi.ac.uk/pride). Fujimoto et al.^93^ performed inhibitor studies and showed that PI 3-kinases are mainly controlling endocytosis of CESAs while PI 4-kinases are involved in exocytosis. The PI 4 kinase At5g64070 is required for proper organization of the TGN and post-Golgi secretion in root hairs. Furthermore, the PI 4 kinase activity is Ca^2+^-dependent.^94^ However, no clear conclusions can be drawn from the phosphorylation pattern observed for this kinase. However, PIP 4 kinase At1g26270, PIP 3/4 kinase At1g49340 and PIP 3 phosphatase At3g19420 exhibit rapid changes in their phosphorylation patterns in response to cellotriose application (Figure 1) and are involved in various vesicle forming events at the plasma membrane. PIP 3/4 kinase, for instance, is located at the plasma membrane and controls autophagosome formation under stress.^95^

Comparison of CESA exocytosis and endocytosis shows that proteins belonging to the first category are mainly phosphorylated while those of the latter category are mainly dephosphorylated. It is tempting to assume that cellotriose stimulates exocytosis and restricts endocytosis to promote cellulose synthesis, however this requires extensive analyses.

### CESA degradation

Plasma membrane protein complexes are typically assembled within the ER and misfolded proteins are targeted to the ER degradation machinery. It is discussed that CESAs form trimeric assemblies shortly after translocation into the ER.^36^ Degradation of misfolded CESAs due to rapid reversible phosphorylation events could not be detected in our study. Different half-life times have been determined for CESAs, ranging from 48 h to 7–8 min, when the proteins are associated with the plasma membrane.^33^ The difference is caused by an efficient recycling of the CSC subunits after endocytosis from the plasma membranes, highlighting again the importance of the endomembrane vesicles for the CESA recycling. Ultimately, degradation of the CESAs follows the ubiquitination pathway.^43,96^ Phosphorylation might play a crucial role in this process: for instance, phosphorylation of CESA7 has been linked to its degradation via the 26S proteasome pathway.^50^ Since we did not identify differentially phosphorylated degradation proteins which have been related to CESAs, it appears that CESA degradation is not controlled by the cellotriose-induced pathway, at least not in the first 15 min after the application of the stimulus.

### Cross-talk to the biosynthesis of other cell wall polysaccharides

Zhang et al.^97^ showed that mutation of CESA1 phosphorylation site on Thr166 influences pectin synthesis and methylesterification. Although this demonstrates a crosstalk between pectin and cellulose biosynthesis, Thr166 CESA1 phosphorylation is not detectable within the first 15 min after cellotriose application. However, to test whether cellotriose application also phosphorylates enzymes involved in pectin or hemicellulose biosynthesis within the first 15 min after application, we analyzed our dataset. No known protein required for hemicellulose biosynthesis was found in our list. For pectin, the pectin methyltransferases At1g78240, At1g53840 and At5g65810 and the pectinerase I At1g53840 were identified. At5g65810 is involved in homogalacturonan pectins methylesterification in the Golgi apparatus prior to integration into cell wall.^98^ Apparently, the cellotriose/CORK1 pathway stimulates mainly cellulose synthesis, although the identified proteins which are not specifically involved in CESA secretion might also be involved in hemicellulose or pectin transport. However, it is also possible that many proteins involved in pectin and hemicellulose biosynthesis are not accessible for kinases/phosphatases within 15 min after the cellotriose stimulus, because they are sequestered in the ER/TGN.

### Cellotriose did not affect the mRNA level for proteins involved in cellulose biosynthesis or protein trafficking

To test whether the cellotriose/CORK1 pathway controls also expression of the genes for the proteins involved in cellulose biosynthesis or protein trafficking, we analyzed expression profiles obtained 1 h, 4 h, and 8 h after the cellotriose stimulus to the roots. None of the mRNA levels for the proteins discussed in this study was > 2-fold regulated in response to cellotriose application. This suggests that cellotriose/CORK1 signaling controls cellulose biosynthesis and protein trafficking preferentially by interfering with the phosphorylation pattern of the involved proteins.

### The cellotriose/CORK1 pathway neither stimulates phosphorylation nor expression of general marker proteins for endomembrane trafficking

Groen et al.^99^ applied and compared multiple approaches to establish a high-confidence data set of Arabidopsis root tissue TGN proteins, in which cargo proteins that are en route to their final cellular destination can be distinguished from full-time endomembrane residents who carry out their function at a given location. Interestingly, none of the 30 proteins, which they defined as TGN marker proteins, were either significantly phosphorylated/dephosphorylated under our conditions nor were their mRNA levels regulated. This suggests that the identified phosphoproteins are specific targets of the cellotriose/CORK1 pathway.

## Conclusion

Phosphorylation of CESAs and their regulatory proteins has been extensively investigated, and analyses of mutants with alterations in the phosphorylation sites demonstrated that phosphorylation is required for CESA activity, movement of CSC within the plasma membrane, as well as trafficking of inactive CESAs from the endomembrane system to the plasma membrane^100^ (summarized in^49^). However, the stimuli and kinases that induce the phosphorylation events are still poorly understood. Besides signals deriving from developmental programs, receptor kinases located at or in the plasma membrane are likely candidates to regulated cellulose biosynthesis in response to external signals. CWI signaling was often associated with *Catharanthus roseus* receptor (like) kinases with MD or MDL domains,^2,29,49,101–105^ among them is FERONIA^29,104^ with its extracellular domain that is recognized by pectic homogalacuronan complexes in the presence of Ca^2+^.^29^ Furthermore, genetic screens identified THESEUS1, another MDL-containing receptor kinase, to be involved in CWI signaling.^103^ WAK1 and so far uncharacterized members of the WAK family interact with pectin in the presence of Ca^2+^.^106,107^ Furthermore, MIK2 (a LRR-receptor kinase MALE DISCOVERER1-INTERACTING RECEPTOR-LIKE KINASE2) might operate upstream of THESEUS1 and alters expression profiles in a CESA6-dependent manner in Arabidopsis.^108^ In addition, FEI1 and FEI2, leucine-rich repeat receptor kinases and SOS5 (SALT OVERLY SENSITIVE5) are involved in cellulose biosynthesis and have been proposed to transduce apoplastic signals into appropriate changes in the cell wall architecture.^109^ Tseng et al.^110^ recently demonstrated that CORK1 is an important MD-RK for many downstream responses associated with CWI signaling in response to cellotriose/cellobiose in the apoplast. Here, we propose that (de-)phosphorylation of crucial proteins involved in cellulose synthesis and those involved in cellular protein trafficking are fast responses to cellotriose. Table 1 demonstrates that many of the phosphorylation events that differ substantially in wild-type and *cork1* mutant roots, indicating that the receptor is involved in cellotriose signaling. However, some of the responses are not CORK1-dependent suggesting that cellotriose may also activate other pathways or require additional proteins besides CORK1. Another interesting observation is that several proteins are rapidly phosphorylated after one time, and no longer phosphorylated or even dephosphorylated at the other time point. Apparently, the response is highly dynamic, and without knowing the exact role of the phosphorylation targets of the individual proteins, an interpretation of the results is purely speculative. The model tries to summarize major proteins identified in this study, which are targeted by cellotriose. It may provide a basis for future studies on signals, which regulate cellulose synthase, and intracellular protein sorting (Figure 1).

The phosphorylation cascade, which leads from CORK1 to an active CSC, remains to be determined. Furthermore, the proposed pathway is integrated into a web of signaling events that all control CWI.^109^ Taking into account that the cell wall is vital for all wall containing organisms, more research is required to understand the regulatory scenario.

Besides control of cellulose biosynthesis, we identified proteins of the endomembrane systems as major phosphorylation targets of cellotriose signals. Several of the targets have been proposed to play crucial roles in vesicle cargo specificity and travel destination (e.g. EPSIN1, MTV1). We propose that cellotriose signaling rapidly interferes with the central trafficking hub where secretory, vacuolar, recycling, and endocytic pathways merge.^72^ Redirection of the vesicles to the appropriate destinations ensure optimal cell wall repair.

## Data Availability

Raw sequences for the GWAS and the transcriptome analysis have been deposited to the NCBI Gene Expression Omnibus (GEO) database (https://www.ncbi.nlm.nih.gov/geo/) with the accession no. GSE197891 and GSE198092, respectively. The mass spectrometry proteomics data have been deposited to the ProteomeXchange Consortium via the PRIDE partner repository (http://www.ebi.ac.uk/pride) with dataset identifier PXD033224.
